# Surgical Management of Bone Metastases in Bladder Cancer: A Single‐Center Retrospective Study

**DOI:** 10.1002/cnr2.70524

**Published:** 2026-03-20

**Authors:** Pavlos Doumanidis, Panagiotis Tsagkozis

**Affiliations:** ^1^ Department of Molecular Medicine and Surgery Karolinska Institutet Stockholm Sweden; ^2^ Karolinska University Hospital Solna Sweden

**Keywords:** bladder cancer, bone metastases, complications, skeletal related events, surgical treatment

## Abstract

**Background and Purpose:**

Bladder cancer (BC) frequently spreads to the bone, resulting in skeletal‐related events with a major effect on quality of life. Since surgical treatment for bone metastases (BM) in BC patients remains understudied, we aimed to evaluate the outcome of surgery in a case series of 20 consecutive patients.

**Patients and Methods:**

A retrospective of BC patients surgically treated for BM at our center between 2007 and 2024. Patient characteristics, surgical procedures, and outcomes, as well as postoperative survival, were analyzed.

**Results:**

Of the 20 patients with BC, who were operated for BM, 14 (70%) were male, with a median age at diagnosis of BM of 72 years. The femur was the most commonly metastatic site (55%), followed by the pelvis (20%) and tibia (15%). The median time between BC diagnosis and surgery for BM was 18 months, and the most frequent event was a complete pathological fracture (70%). Prosthetic joint reconstruction was used for 10 (50%) of our patients, making it the most commonly used procedure in our cohort. Seven patients experienced postoperative complications, and two underwent reoperation. Median postoperative survival was 2 months, and only two patients were alive at last follow‐up, both of them presenting with BM many years after initial BC diagnosis.

**Conclusion:**

Most BM in BC are in the lower extremity and pelvis and are lytic in character. The oncological outcome is poor, with short postoperative survival. The choice of surgical method should take into account the generally palliative stage of the disease. Patients presenting with late BM may have a better prognosis and thus benefit from more advanced surgical reconstruction. Surgery can provide symptomatic relief and structural stabilisation, but the risk for postoperative complications is considerable.

## 1 Introduction

Bladder cancer (BC) ranks as the 10th most commonly diagnosed cancer worldwide, holding the 7th place among males, and being the 13th leading cause of cancer‐related mortality [1]. Around 25% of BC cases present as muscle‐invasive or metastatic disease, with bone being the most frequent site of distant metastases apart from the lymph nodes, and bone metastases (BM) in BC are associated with poor prognosis [2, 3]. Patients with bone‐metastatic BC actually have the worst survival among genitourinary malignancies with BM [4, 5].

BM are associated with skeletal‐related events (SREs), such as radiation therapy, surgery for pathological fractures or spinal cord compression, and hypercalcemia, leading to significantly reduced quality of life [6]. Metastatic bone disease and associated pathological fractures, completed or impending, are often treated surgically, in order to restore the ambulatory capacity and alleviate pain. Furthermore, surgery may be needed in cases of neurological compromise due to spinal metastatic disease. There is however significant variability in the manifestations of BM among cancer patients, depending on the primary diagnosis and extent of the disease. The choice of surgical method is largely dependent on such data, which also entails a prognosis regarding survival. Previous studies have described the results of surgical treatment for pathological fractures in both common diagnoses, such as breast and prostate cancer, and in some less common primary tumours, to aid surgeons and patients in informed decision‐making [7, 8, 9].

Although BM are relatively common among BC patients, their surgical treatment has only been described in case reports [10, 11]. The lack of information on the outcome of orthopedic surgery in BC patients with BM hinders surgeons in choosing the proper reconstruction method. Furthermore, patients' counseling regarding the expected results and complications of surgical treatment and the overall prognosis, including survival, is severely restricted by the absence of published data. The current study seeks to cover this knowledge gap. Our primary aim was to describe the oncological outcome in terms of postoperative survival. The secondary aim was to describe the surgical outcome including function and complications.

## 2 Patients and Methods

### 2.1 Study Design

This is an institutional retrospective review of medical records of ΒC patients who underwent surgical treatment for BM between 2007 and 2024. The institutional database was used to retrieve primary data, and patient files were further checked to confirm the validity and completeness with other information when required. The primary endpoints were overall patient survival measured from surgery to date of death from any cause, and local or systemic complications. Other parameters analysed were: age of patient at first surgery, sex, location of BM, time from BC diagnosis to surgery for BM, reason for surgery, method of surgical reconstruction, and any reoperation. All patients included in this study underwent surgical treatment at the Department of Orthopedics, Karolinska University Hospital, Stockholm, Sweden. Incomplete medical records or a lack of follow‐up data were the exclusion criteria.

We identified 20 cases of BC patients surgically treated for BM from 2007 to 2024 among a total of 614 BM patients in the database. All patients were included in the analysis. The study was conducted according to the ethical permits 2012/272–31 and 2019–06189 of the Regional Ethics Committee in Stockholm. Sweden. The patient consent was waived due to the retrospective nature of the study. There was no use of Artificial Intelligence.

### 2.2 Statistical Analysis

Descriptive statistics were used to describe clinicopathological characteristics. Continuous variables were presented as medians with interquartile ranges (IQRs), while categorical variables were expressed as frequencies and percentages. To evaluate survival, the Kaplan–Meier method was used, with median survival and 95% confidence intervals (CIs) reported. Cox‐regression survival analysis was performed to evaluate the effect of time from cancer diagnosis to BM surgery on overall patient survival. All statistical analyses were conducted in the SPSS software (version 29.0, IBM Corp., NY, USA).

## 3 Results

### 3.1 Patient Demographics and Oncological Outcome

The median age at surgery was 72 years, and males represented 70% of the cases (*n* = 14). Four patients presented with BM, while for the rest, the median period from BC diagnosis to bone metastasis surgery was 18 (range: 2–120) months. The median period from symptom onset, indicating the beginning of metastasis development, to surgery was 2 months (range: 0.5–18 months). In 10 individuals, BM was the only sign of metastasis, while in the other 10 patients, BM was discovered after additional visceral metastases had been diagnosed. Patient demographics are presented in Table 1.

**TABLE 1:** Patient demographic characteristics, including age and gender, location of bone metastases, and spread of the disease.

	Value, *n* (%)
Number of patients	20
Age at surgery (years), mean ± standard deviation	72 ± 1
Sex	
Female	6 (30%)
Male	14 (70%)
Skeletal metastases	
Multiple lesions	10 (50%)
Solitary lesion	8 (40%)
Unknown	2
Other metastases	
Lymph node	7 (35%)
Lung	5 (25%)
Liver	1 (5%)
Location of bone metastases	
Femur	11 (55%)
Pelvis	4 (20%)
Humerus	2 (10%)
Femur and tibia	2 (10%)
Spine	2 (10%)
Tibia	1 (5%)

Regarding referral pathways, 11 patients sought to an emergency department due to an acute onset of symptoms, eight were electively referred from the treating oncologist or urologist with a recommendation to consider surgery, and one was referred from the general practitioner. The indication for surgery took into account the patients' symptoms, general condition, and expected survival, and the final decision was taken by the treating orthopedic surgeon after thorough discussion with the patient.

Prior chemotherapy had been administered in eight patients (4 had gemcitabine/cisplatin, 3 gemcitabine/carboplatin, and 1 gemcitabine monotherapy). Prior local radiotherapy had been administered to three patients. Two patients had bisphosphonates preoperatively (one pamidronate, one alendronate), and another one had denosumab.

**FIGURE 1:**
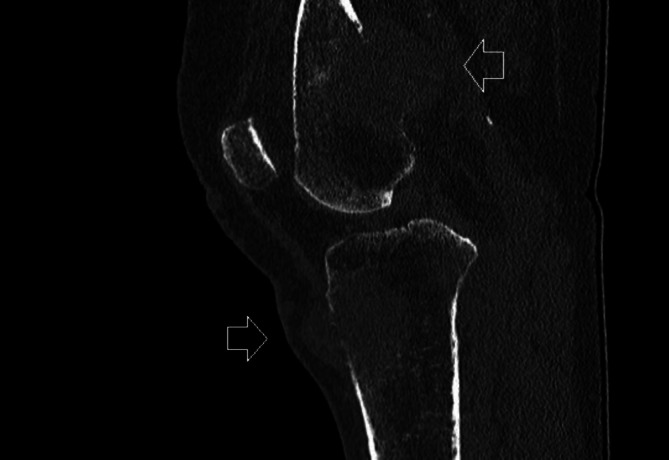
Preoperative radiograph of the knee (lateral view) demonstrating lytic metastatic lesions of both the proximal tibia and the distal femur with cortical destruction.

**FIGURE 2:**
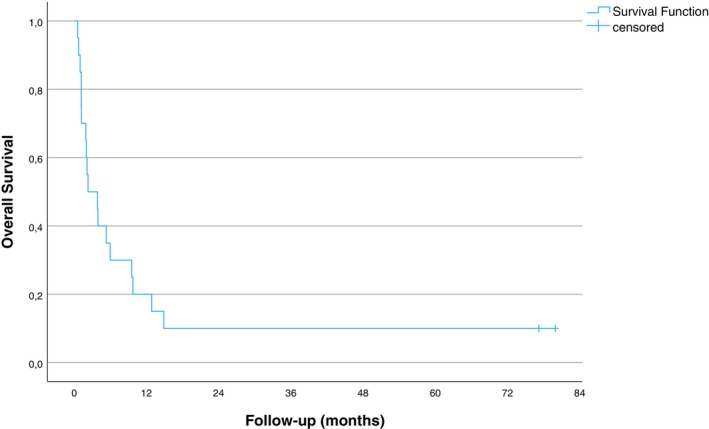
Kaplan–Meier curve showing overall survival following surgical treatment for bone metastases in bladder cancer patients.

The femur was the most common site for BM, present in 11 patients (55%), two of those with concomitant tibia BM (Figure 1). This was followed by the pelvis in four patients (20%), tibia in three patients (15%), and humerus and spine in two patients (10%). The most frequently observed event was a complete fracture, occurring in 14 patients (70%). Impending fractures were identified in four patients (20%), while two patients (10%) had neurological compromise due to spinal lesions. Metastatic lesions were predominantly lytic in character. Most of our patients (*n* = 18) were dead at the last follow‐up. The median postoperative survival time was 2 months (95% CI, 1–6 months) (Figure 2). Both patients alive at follow‐up were long‐term survivors (6.5 years of postoperative follow‐up each), and both presented with BM long time after diagnosis of the BC (6.5 and 10 years). In Cox‐regression analysis, time from BC diagnosis to surgery was significantly associated with overall postoperative survival (*p* = 0.043).

### 3.2 Surgical Treatment, Complications, and Reoperations

Since most lesions were near the joints of the long bones, joint replacement (arthroplasty) was the technique used most often, with 10 patients (50%) undergoing it (Table 2). The next most common surgical reconstruction method was bone fixation (osteosynthesis with either intramedullary nails or plate and screws), which was performed in five patients. Spinal surgery (decompressive laminectomy with or without instrumentation) was performed in two patients, whereas one patient underwent amputation, one curettage and cement augmentation, and one local pelvic excision. Most operations (17/20) involved limited or no excision of the cancerous tissue at the site of the lesion. Only in three cases was there a radical excision of the BM.

**TABLE 2:** Surgical treatment.

	Value, *n* (%)
Surgical reconstruction	
Joint reconstruction (arthroplasty)	10 (50%)
Bone fixation (osteosynthesis)	5 (25%)
Excision without reconstruction	3 (15%)
Spinal surgery	2 (10%)

Five patients experienced complications. Three of them had surgical complications, of whom two were noted in the immediate postoperative period: a deep infection in one patient, and vessel injury and bowel perforation in another. One patient had a late local complication, since he sustained a secondary fracture near a lesion who had previously been curetted and augmented with bone cement, and was subsequently treated with a cast. Medical complications were noted in two patients, and were recorded in the early postoperative period (one patient had a urinary tract infection and another a deep venous thrombosis and sepsis). Local reoperation was performed in two patients: one who had replacement of a malpositioned implant, while in another one, who had previously been operated on with an intramedullary nail, lesion progression was observed, and the fixation was converted to plate and screw osteosynthesis, but finally resulted in amputation due to further local tumor progression.

## 4 Discussion

In this retrospective study, we examined the clinical features, therapeutic approaches, and postoperative outcome and survival of 20 bc patients with BM treated in our clinic. The current study has several limitations. First, there is a small sample size, and it is retrospective in nature, limiting the statistical power, not allowing multivariable analysis, and restricting the generalizability of the findings. Selection bias in treatment may also limit the interpretation of the results. The study analyzes the outcome of patients selected for surgical treatment, and there is no comparison to patients treated non‐surgically, which also limits the interpretation of the results to this particular patient group. However, it is the first published cohort of BC patients operated for BM, since there have only been case reports thus far (10, 11). Our study can thus aid general practitioners and urologists in the timely diagnosis and expected clinical outcome, and orthopedic surgeons in the better choice of surgical treatment method.

Bone metastases occur in 30%–40% of patients with metastatic BC [12]. A number of characteristics, such as being between the ages of 41 and 60 and lung, liver, and brain metastases, are associated with BM [5]. The patients in our cohort were older, with a median age of 72 years, which may be due to the fact that older patients are more prone to pathological fractures needing surgical treatment.

We observed that the most common site was the femur, followed by pelvis. These results are consistent with other studies that found that weight‐bearing bones, especially the femur and pelvis, which are highly vascularized and structurally susceptible to metastatic involvement, have a predilection for BM [13]. The fact that pelvic lesions were not predominant in our cohort is probably due to the fact that only a few of them are treated surgically, namely, the ones near the acetabulum. In any case, our findings call for high clinical suspicion and early radiological workup in BC patients with hip pain. Notably, two of our patients with distal femoral lesions had lesions in the tibia, raising the interesting possibility that the cancer cells spread through the synovial tissue or fluid to the opposite site of the joint. Interestingly, there have been two case reports of synovial metastasis from BC, which implies that this neoplasm has a rare propensity to home to the synovial tissue [14, 15].

Bone metastases in BC are associated with a higher rate of SREs, such as spinal cord compression and pathological fractures, which significantly reduce quality of life and functional status. The most frequent SREs in our group were non‐spinal complete fractures. The surgical treatment of our patients was customized according to the general health status, the location, size of the lesions, degree of bone loss, and the functional status. For instance, a prosthesis was frequently used to relieve pain and restore function in lesions near the big joints, such as proximal femoral lesions. Similarly, intramedullary nailing was used to stabilize and facilitate early mobilization in cases of diaphyseal fractures. When deciding on the best treatment options, the estimated survival time is important for the selection of the most suitable surgical method [16]. Our cohort's median OS of 2 months is consistent with other studies that found that BC patients with BM had a poor prognosis [4, 5, 17]. Interestingly, we could document a clear association between the time elapsed from initial cancer diagnosis to surgery for BM, and overall postoperative survival. A similar effect was also observed in a population‐based study [18]. The correlation should be taken into account, and aggressive surgical reconstruction should only be reserved for patients with good general condition, preferably those presenting with BM many years after the initial BC diagnosis. Physicians and patients should also be aware of the considerable number of surgical and general complications associated with surgery, which is, however, necessary in most such lesions in order to restore functional deficits and alleviate pain.

## Author Contributions

**Pavlos Doumanidis:** data curation, formal analysis, investigation, writing‐original draft. **Panagiotis Tsagkozis:** resources, supervision, formal analysis, project administration, writing – review and editing, validation, visualization, conceptualization, funding acquisition, methodology.

## Funding

The authors have nothing to report.

## Conflicts of Interest

The authors declare no conflicts of interest.

## Data Availability

The data that support the findings of this study are available on request from the corresponding author. The data are not publicly available due to privacy or ethical restrictions.

## References

[1] H. Sung , J. Ferlay , R. L. Siegel , et al., “Global Cancer Statistics 2020: GLOBOCAN Estimates of Incidence and Mortality Worldwide for 36 Cancers in 185 Countries,” CA: A Cancer Journal for Clinicians 71, no. 3 (2021): 209–249. 33538338 10.3322/caac.21660

[2] M. Burger , J. W. Catto , G. Dalbagni , et al., “Epidemiology and Risk Factors of Urothelial Bladder Cancer,” European Urology 63, no. 2 (2013): 234–241. 22877502 10.1016/j.eururo.2012.07.033

[3] A. B. Smith , A. M. Deal , M. E. Woods , et al., “Muscle‐Invasive Bladder Cancer: Evaluating Treatment and Survival in the National Cancer Data Base,” BJU International 114, no. 5 (2014): 719–726. 24325202 10.1111/bju.12601

[4] T. Owari , M. Miyake , Y. Nakai , et al., “Clinical Features and Risk Factors of Skeletal‐Related Events in Genitourinary Cancer Patients With Bone Metastasis: A Retrospective Analysis of Prostate Cancer, Renal Cell Carcinoma, and Urothelial Carcinoma,” Oncology 95, no. 3 (2018): 170–178. 29874673 10.1159/000489218

[5] C. Zhang , L. Liu , F. Tao , et al., “Bone Metastases Pattern in Newly Diagnosed Metastatic Bladder Cancer: A Population‐Based Study,” Journal of Cancer 9, no. 24 (2018): 4706–4711. 30588255 10.7150/jca.28706 PMC6299390

[6] Y. Tsuda , T. Nakagawa , Y. Shinoda , et al., “Skeletal‐Related Events and Prognosis in Urothelial Cancer Patients With Bone Metastasis,” International Journal of Clinical Oncology 22, no. 3 (2017): 548–553. 28044212 10.1007/s10147-016-1075-9

[7] R. J. Weiss , J. A. Forsberg , and R. Wedin , “Surgery of Skeletal Metastases in 306 Patients With Prostate Cancer,” Acta Orthopaedica 83, no. 1 (2012): 74–79. 22206449 10.3109/17453674.2011.645197 PMC3278661

[8] A. Fottner , M. Szalantzy , L. Wirthmann , et al., “Bone Metastases From Renal Cell Carcinoma: Patient Survival After Surgical Treatment,” BMC Musculoskeletal Disorders 11 (2010): 145. 20598157 10.1186/1471-2474-11-145 PMC2909163

[9] R. Wedin , H. C. Bauer , and L. E. Rutqvist , “Surgical Treatment for Skeletal Breast Cancer Metastases: A Population‐Based Study of 641 Patients,” Cancer 92, no. 2 (2001): 257–262. 11466677 10.1002/1097-0142(20010715)92:2<257::aid-cncr1317>3.0.co;2-r

[10] T. Jouma Alhejazi , H. Bdeiwi , M. W. Sukkari , M. Ibrahim , A. Sukari , and H. Alloush , “Femoral Metastasis in Previously Treated Bladder Cancer Patient: A Case Report,” Clinical Case Reports 10, no. 9 (2022): e6357. 36177084 10.1002/ccr3.6357 PMC9474905

[11] M. H. Mohammed , F. Mardnly , M. Ghrer , L. Alia , and L. W. Assad , “Unusual Metastasis After Radical Cystectomy: Case Report,” Journal of Surgical Case Reports 2024, no. 3 (2024): rjae112. 38455984 10.1093/jscr/rjae112 PMC10918444

[12] A. B. Shinagare , N. H. Ramaiya , J. P. Jagannathan , F. M. Fennessy , M. E. Taplin , and A. D. Van den Abbeele , “Metastatic Pattern of Bladder Cancer: Correlation With the Characteristics of the Primary Tumor,” AJR. American Journal of Roentgenology 196, no. 1 (2011): 117–122. 21178055 10.2214/AJR.10.5036

[13] M. Stellato , D. Santini , M. C. Cursano , S. Foderaro , G. Tonini , and G. Procopio , “Bone Metastases From Urothelial Carcinoma. The Dark Side of the Moon,” Journal of Bone Oncology 31 (2021): 100405. 34934613 10.1016/j.jbo.2021.100405 PMC8661045

[14] R. Khurram , A. Khurram , and K. Chaudhary , “Index Case of Synovial Metastasis in a Patient With Transitional Cell Carcinoma of the Bladder,” BML Case Reports 13, no. 6 (2020): e235084. 10.1136/bcr-2020-235084 PMC7322511 32595116

[15] R. T. S. Wang , Q. Zhuang , and X. F. Chen , “Synovial Metastasis From Urothelial Carcinoma of the Renal Pelvis Causing Recurrent Hemarthrosis: A Rare Presentation,” Cureus 15, no. 3 (2023): e36983. 37139285 10.7759/cureus.36983 PMC10149888

[16] H. Katagiri , R. Okada , T. Takagi , et al., “New Prognostic Factors and Scoring System for Patients With Skeletal Metastasis,” Cancer Medicine 3, no. 5 (2014): 1359–1367. 25044999 10.1002/cam4.292 PMC4302686

[17] F. Dong , Y. Shen , F. Gao , et al., “Prognostic Value of Site‐Specific Metastases and Therapeutic Roles of Surgery for Patients With Metastatic Bladder Cancer: A Population‐Based Study,” Cancer Management and Research 9 (2017): 611–626. 29180897 10.2147/CMAR.S148856 PMC5694197

[18] A. A. Nelson , R. J. Cronk , E. A. Lemke , et al., “Early Bone Metastases Are Associated With Worse Outcomes in Metastatic Urothelial Carcinoma,” Bladder Cancer 7, no. 1 (2021): 33–42. 38993215 10.3233/BLC-200377 PMC11181800

